# The association between gender equality and climate adaptation across the globe

**DOI:** 10.1186/s12889-024-18880-5

**Published:** 2024-05-24

**Authors:** Ana-Catarina Pinho-Gomes, Mark Woodward

**Affiliations:** 1 The George Institute for Global Health, Imperial College London, Scale Space, 58 Wood Lane, London, W12 7RZ UK; 2 Institute for Global Health, University College London, London, UK; 3 The George Institute for Global Health, University of New South Wales, Sydney, Australia

**Keywords:** Gender inequalities, Climate change, Climate adaptation

## Abstract

**Introduction:**

Climate change has a disproportionate impact on women in comparison to men, and women have a key role to play in climate adaptation. However, evidence is lacking on how gender inequalities may be associated with climate vulnerability and ability to respond at country level.

**Methods:**

This ecological study investigated the association between climate adaptation, measured by the Notre Dame Global Adaptation Initiative Country Index (ND-GAIN), and gender equality, measured by the Global Gender Gap Index (GGGI) developed by the World Economic Forum and the Gender Inequality Index (GII) developed by the United Nations. Simple linear regression was used to estimate the associations between the indices and their subdomains for 146 countries.

**Results:**

There was an approximately linear association between the GGGI and climate adaptation. Each 1% increase in gender equality was associated with a 0.6% increase in the ND-GAIN score (the slope was 0.59, with a 95% confidence interval [0.33 to 0.84]). This was driven by a negative association between gender equality and vulnerability (-0.41 [-0.62 to -0.20]), and a positive association between gender equality and readiness (0.77 [0.44 to 1.10]). The strongest associations between gender equality and climate adaptation were observed for the education domain of the GGGI. There was a strong negative linear association between the GII and climate adaptation, which explained most (86%) of the between-country variation in climate adaptation. Each 1% increase in gender inequality was associated with a 0.5% decrease in the ND-GAIN score (-0.54 [-0.57 to -0.50]). The association between gender inequality and readiness was stronger than the association with vulnerability (0.41 [0.37 to 0.44] for vulnerability versus − 0.67 [-0.72 to -0.61] for readiness).

**Conclusions:**

Gender inequality, measured broadly across different domains of life, is associated with climate adaptation at country level, both in terms of vulnerability to impact and readiness to respond.

**Supplementary Information:**

The online version contains supplementary material available at 10.1186/s12889-024-18880-5.

## Introduction

The climate crisis, as with many other crises including the COVID-19 pandemic, is not gender neutral. On the one hand, women and girls across the globe experience the greatest impact of climate change and have the least capacity to respond to climate-related hazards, such as heatwaves, floods, volcanic eruptions, and hurricanes [1–4]. On the other hand, women’s unequal participation in policy, decision making and labour markets prevents them from fully contributing to climate change mitigation [5]. Barriers to women’s participation include time constraints due to other responsibilities, such as childcare, and lack of experience, awareness, support within institutional frameworks, and access to finance [6]. This has been acknowledged by the United Nations Framework Convention on Climate Change (UNFCCC), which established a dedicated agenda item on gender and climate change under the Convention and included overarching text in the Paris Agreement [7]. 

Climate change has a disproportionate impact on women, in comparison to men, particularly in low- and middle-income countries (LMIC). Worldwide, populations that rely on natural resources for their livelihoods, as well as those experiencing poverty, are the most vulnerable to climate change. Women are over-represented in both groups [5]. Furthermore, climate-induced extreme weather events are forcing migration and causing conflicts, with the UN estimating that 80% of people displaced by climate change are women [5]. Climate migration and extreme events expose women to increased risk of gender-based violence and trafficking due to economic instability, food insecurity, mental stress, disrupted infrastructure, increased exposure to men [8, 9]. Climate change is, thus, a threat multiplier for women’s health and wellbeing.

Women have a huge potential to mitigate, adapt, and respond to climate change [10]. Particularly in LMICs, women’s local knowledge and leadership has been critical for implementation of sustainable practices at household and community level. For instance, in South Africa, local women farmers are trained on agroecology and permaculture farming to overcome challenges posed by erratic weather conditions and climate change impacts [11]. This provides them with unique knowledge and skills that can help make the response to climate change more effective and sustainable. It also helps them to be more respected and given an opportunity in the current male-dominated positions. Globally, companies with a greater share of women in boards are more likely to adopt climate and eco-friendly policies [12]. Likewise, countries with a larger representation of women in Parliament are more prone to ratify environmental treaties and adopt policies that address climate change [13]. However, women still experience barriers in ascending to positions of leadership in government and private companies, and hence remain underrepresented in both settings [14, 15]. 

Considering both women’s disproportionate vulnerability and their role in the response to climate change, it is germane to ask to what extent gender inequality may exacerbate the impact and undermine collective efforts to adapt to climate change. Therefore, this study aimed to investigate the association between gender inequality in several societal domains and vulnerability and readiness to adapt to climate change across the globe. This evidence may reinforce the importance of gender equality for successful climate adaptation. Conversely, it could support the argument that failure to adapt to climate change may further exacerbate gender inequality.

## Methods

We conducted an ecological study to investigate the association between gender equality and climate adaptation across the globe.

### Gender equality

We used the Global Gender Gap Index (GGGI), developed by the World Economic Forum, and the United Nations Gender Inequality Index (GII), to assess gender equality. The GGGI was first introduced in 2006 to benchmark progress towards gender parity and compare countries’ gender gaps across four dimensions: economic opportunities, education, health, and political leadership. There are three basic concepts underlying the GGGI, forming the basis of how indicators were chosen, how the data are treated and how the scale can be used. First, the index focuses on measuring gaps, rather than levels. Second, it captures gaps in outcome variables, rather than gaps in input variables. Third, it ranks countries according to gender equality, rather than women’s empowerment. The GGGI includes four subindexes, each of which is calculated from several indicators (Table S1). The overall GGGI score is a simple average of each subindex score and, similarly to subindex scores, ranges between one (parity) and zero (imparity). Detailed information about the GGGI is available elsewhere [16]. 

The GII is a composite metric that reflects gender-based disadvantage in three dimensions: reproductive health, empowerment, and the labour market. It shows the loss in potential human development due to inequality between women and men’s achievements in these dimensions. It ranges from zero (parity) to 1 (imparity). The GII values are computed using several indicators (Table S2) [17]. A detailed description of the GII is available elsewhere [18]. Note that, contrary to the GGGI, higher values of GII denote greater gender equality.

### Climate vulnerability and readiness

We used the Notre Dame Global Adaptation Initiative (ND-GAIN) Country Index to assess climate adaptation worldwide. The ND-GAIN Country Index is composed of two key dimensions of adaptation: vulnerability and readiness (Table 1). Vulnerability reflects the exposure, sensitivity, and capacity of each country to adapt to the negative effects of climate change. The index considers overall vulnerability across six life-supporting sectors: food, water, health, ecosystem service, human habitat and infrastructure. Readiness reflects the ability of each country to leverage investments and convert them into adaptation actions. The index considers overall readiness across three components: economic readiness, governance readiness, and social readiness. Both vulnerability and readiness vary between zero and one, but whilst lower is better for vulnerability, higher is better for readiness. Thirty-six indicators are used to assess vulnerability and nine indicators measure aspects of readiness. The overall ND-GAIN Score is calculated as the average of the vulnerability and readiness scores for each country and varies between zero and one (higher is better adaption). An explanation of each indicator and their data sources is available elsewhere [19]. 

**Table 1:** Domains included in the vulnerability and readiness dimensions of the ND-GAIN Index

**Vulnerability**	Exposure: degree to which a system is exposed to significant climate change from a biophysical perspective. It is a component of vulnerability independent of socio-economic context. Exposure indicators are projected impacts for the coming decades and are therefore invariant over time.
	Sensitivity: extent to which a country is dependent upon a sector negatively affected by a climate hazard, or the proportion of the population particularly susceptible to a climate change hazard. A country’s sensitivity can vary over time.
	Adaptive capacity: availability of social resources for sector-specific adaptation. In some cases, these capacities reflect sustainable adaptation solutions. In other cases, they reflect capacities to put newer, more sustainable adaptations into place. Adaptive capacity varies over time.
**Readiness**	Economic: captures the ability of a country’s business environment to accept investment that could be applied to adaptation that reduces vulnerability (reduces sensitivity and improves adaptive capacity).
	Governance: captures the institutional factors that enhance application of investment for adaptation.
	Social: captures the factors, such as social inequality, ICT infrastructure, education, and innovation, that enhance the mobility of investment and promote adaptation actions.

### Data analysis

To ensure that all data used are contemporaneous we used data from 2022 for all three indices, this being determined by the GII index, which had the least recent data available. For ease of interpretation, both the GGGI, the GII, and the ND-GAIN indices, and respective subindices, were converted into percentages by multiplying the scores by 100. Whilst maintaining the relationship between the indices, conversion of proportions into percentages made the indices easier to understand. Countries were grouped into regions according to the World Bank classification [20]. Simple linear regression was used to estimate the association between the GGGI, and its subindices, and the ND-GAIN index and the vulnerability and readiness dimensions. Scatterplots were used to display the associations with loess (i.e., local polynomial regression) curves fitted to explore the inherent assumption of linearity. We also estimated the correlation between the GGGI and GII using Spearman’s correlation coefficient (r). All analyses were carried out using R version 4.3.0.

## Results

### Worldwide distribution of the ND-GAIN, GGGI and GII

**Fig. 1:**
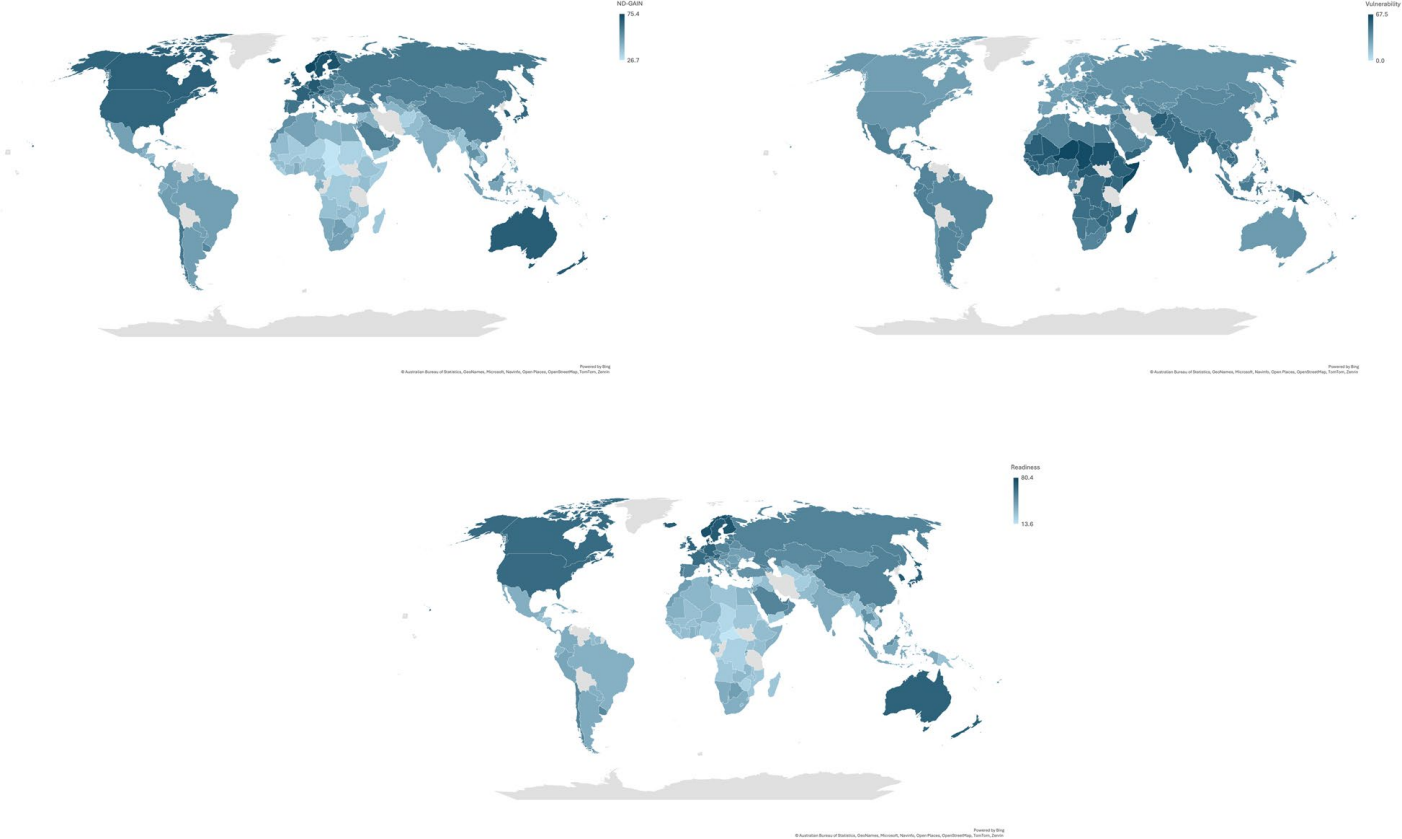
World map illustrating the distribution of the Notre Dame Global Adaptation Index (ND-GAIN), Global Gender Gap Index (GGGI) and Gender Inequality Index (GII)

A total of 146 countries were included in this study (details are available in Table S3). The median GGGI was 71% with a range from 43% for Afghanistan to 91% for Iceland. The median GII was 33% and it ranged from 1% Denmark to 82% for Yemen. The median of the ND-GAIN score was 50% and it varied between 27% for Chad and 75% for Norway. There was more variation in readiness than vulnerability (median of vulnerability 41%, range 25% for Switzerland to 67% for Niger, and median of readiness 43%, range 19% for Central African Republic to 80% for Singapore). In these data there was a modest correlation between the GII and GGGI (*r* = -0.31). In general, countries in Europe and North America tended to cluster at the lower end of the vulnerability and gender inequality, whilst countries in Africa and South Asia clustered at the higher end of the vulnerability and gender inequality (Fig. 1 and Figure S1).

### Association between the GGGI and the ND-GAIN

**Fig. 2:**
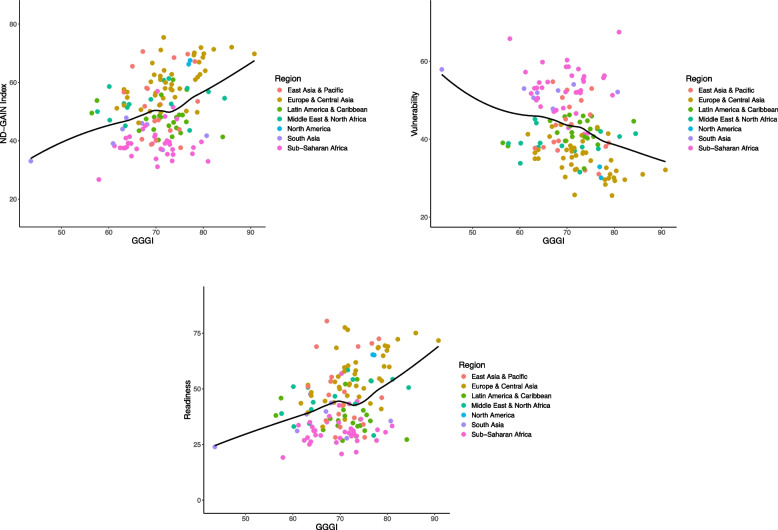
Association between gender equality based on the GGGI and climate adaptation based on the ND-GAIN country index

There was an approximately linear association between gender equality measured by GGGI and climate adaptation (Fig. 2; Table 2). Overall, each 1% increase in gender equality was associated with a 0.6% increase in the ND-GAIN score (the slope was 0.59, with a 95% confidence interval [0.33 to 0.84]). This was driven by a negative association between gender equality and vulnerability, with each 1% increase in gender equality associated with a 0.4% decrease in vulnerability (-0.41 [-0.62 to -0.20]), and a positive association between gender equality and readiness, with each 1% increase in gender equality associated with a 0.8% increase in readiness (0.77 [0.44 to 1.10]). Overall, the strongest associations between gender equality and climate adaptation were observed for the education domain of the GGGI. Each 1% increase in gender equality in the education domain was associated with a 0.4% increase in climate adaptation (0.39 [0.19 to 0.60]). There was a weaker association with gender equality in the economic and political domains (0.21 [0.06 to 0.36] for the economic domain and 0.18 [0.07 to 0.28] for the political domain). There was no evidence of an association between health equality and climate adaptation. The education domain had the strongest association with both vulnerability and readiness (-0.32 [-0.48 to -0.15] for vulnerability and 0.47 [0.20 to 0.74] for readiness).

**Table 2:** Association between gender equality and climate adaptation: regression slopes per percentage change in greater gender equality

Gender equality index/climate adaption index	Estimate [95%CI]	*p* value	*R*^2^
**Global gender gap index (GGGI): higher values mean greater gender equity**			
**ND-GAIN**	0.59 [0.33 to 0.84]	< 0.001	0.118
**Vulnerability**	-0.41 [-0.62 to -0.20]	< 0.001	0.086
**Readiness**	0.77 [0.44 to 1.10]	< 0.001	0.120
**GGGI-Economy**			
**ND-GAIN**	0.21 [0.06 to 0.36]	0.007	0.044
**Vulnerability**	-0.14 [-0.26 to -0.01]	0.030	0.025
**Readiness**	0.29 [0.09 to 0.48]	0.004	0.048
**GGGI-Education**			
**ND-GAIN**	0.39 [0.19 to 0.60]	< 0.001	0.082
**Vulnerability**	-0.32 [-0.48 to -0.15]	0.001	0.083
**Readiness**	0.47 [0.20 to 0.74]	< 0.001	0.068
**GGGI-Politics**			
**ND-GAIN**	0.18 [0.07 to 0.28]	0.001	0.064
**Vulnerability**	-0.12 [-0.20 to -0.03]	0.008	0.041
**Readiness**	0.24 [0.10 to 0.38]	0.001	0.068
**GGGI-Health**			
**ND-GAIN**	-1.08 [-3.05 to 0.90]	0.287	0.001
**Vulnerability**	0.93 [-0.66 to 2.51]	0.254	0.002
**Readiness**	-1.22 [-3.78 to 1.34]	0.351	-0.001
**Gender inequality index (GII): lower values mean greater gender equity**			
**ND-GAIN**	-0.54 [-0.57 to -0.50]	< 0.001	0.860
**Vulnerability**	0.41 [0.37 to 0.44]	< 0.001	0.768
**Readiness**	-0.67 [-0.72 to -0.61]	< 0.001	0.784

*ND-GAIN* Notre Dame Global Adaption Initiative Country Index

### Association between the GII and the ND-GAIN

**Fig. 3:**
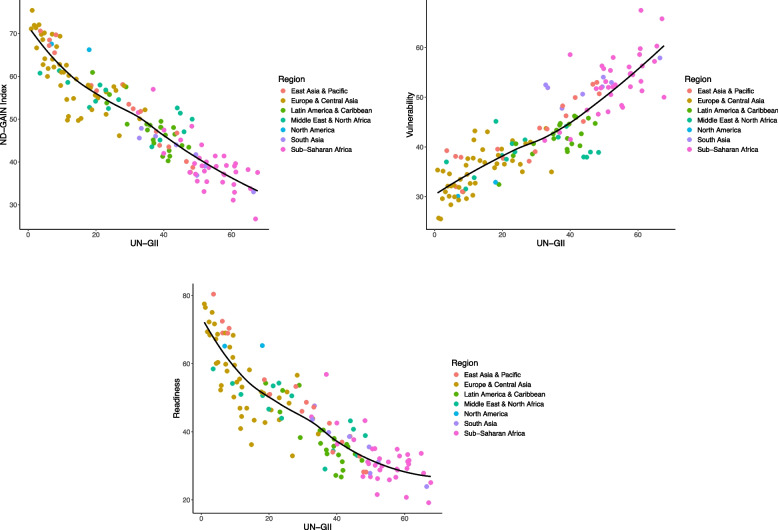
Association between gender equality based on the UN-GII and climate adaptation based on the ND-GAIN country index

When gender inequality was measured by the GII, there was a strong negative linear association between gender inequality and climate adaptation, which explained most (86%) of the between-country variation in climate adaptation (Fig. 3; Table 2). Each 1% increase in gender inequality was associated with a 0.5% decrease in the ND-GAIN score (-0.54 [-0.57 to -0.50]). The association between gender inequality and readiness was stronger than the association with vulnerability (0.41 [0.37 to 0.44] for vulnerability versus − 0.67 [-0.72 to -0.61] for readiness).

## Discussion

This study found an approximately linear association between gender equality, measured using two different indices, and climate adaptation across 146 countries. The unexplained country-to-country variation was considerably less for the GII index than the GGGI. The association was underpinned by a positive association between gender equality and readiness and a negative association between gender equality and vulnerability, irrespective of the index used to assess gender equality. The association with readiness appeared to be stronger than the association with vulnerability.

### Interpretation in light of previous evidence

The underlying reasons for the association between gender inequality and climate adaptation are likely manifold. First, countries with the greatest gender inequality tend to have low socioeconomic development [21]. Therefore, these countries are likely to rely on sectors that are particularly susceptible to the impact of climate change, such as agriculture [22]. In LMICs, about 70% of the estimated 1.3 billion people living below the poverty line are women and most depend on subsistence agriculture to survive [5]. These sectors are also where women are disproportionately employed due to a complex interplay between gendered societal roles and norms and lack of access to education, which then compromises their employment opportunities. This means that gender inequality in education, labour, and economic domains results in women being the most vulnerable population in the most vulnerable countries [23]. Second, the impact of climate change-induced extreme weather events tends to be more severe for women than men [24]. This results from a complex interaction of biological and socioeconomic factors. The increasingly frequent extreme weather events of the past two decades, such as floods, storms, volcanic eruptions, heatwaves, and droughts, have resulted in socioeconomic instability, breakdowns in safety and law enforcement, and political unrest or even armed conflicts. These, in turn, have increase gender-based violence and sexual exploitation, particularly in the context of forced migration [23]. In addition, women are more likely to skip meals as they are often last in household food hierarchies, thus being more exposed to the impact of food scarcity. Gender inequality thus compounds the impact of extreme weather events by further increasing women’s vulnerability and disadvantage.

### Implications for climate policy

Women account for about 50% of the world’s population and hence, from a purely numerical perspective, by excluding women from the workforce and participation in society, politics, and the labour market countries lose substantial human resources. This curtails their ability to respond to the challenges posed by climate change and protect lives and livelihoods, especially in resource-poor settings. Furthermore, women’s contribution to society as well as political and economic systems has wide benefits. For instance, women in positions of authority tend to resolve crises without resorting to violence [25]. Women’s involvement in politics is crucial to assuage tensions that may be precipitated, for instance, by food and water scarcity and vector-borne diseases. Women leaders are also more likely than men to consider and address social injustice and inequalities, which are exacerbated by climate change [10]. Greater representation of women in parliaments and governments increases commitment to sustainability and climate change action [13]. Therefore, fair involvement of women at all levels of societal life, including particularly in leadership positions, is crucial to ensure that countries successfully and equitably adapt to climate change.

Notwithstanding the evidence supporting the importance of involving women in climate adaptation worldwide, women still experience barriers to engaging with climate adaptation, such as gender bias and discrimination, lack of access to financing and education, or gendered roles and responsibilities in the household, community and labour markets [15, 26]. For instance, the relocation of communities affected by raising sea levels in the Fiji islands compelling illustrates how failure to involve women in decision making can compromise climate adaptation [27]. Women reported that men decided they had to relocate and the women had to agree without even being consulted. Their testimonies demonstrated how climate change is exacerbating gender inequalities by perpetuating gendered cultural and societal norms, which often exclude women from decision making [28]. This precludes their full engagement in the response to climate change at local and international levels.

In general, gender equality in leadership enhances the diversity of perspectives, which then leads to more fact-based and, therefore, higher-quality decision-making. There is also compelling evidence that gender equality drives economic prosperity [29]. For instance, the losses to an economy from economic disempowerment of women are estimated to range from 10% of gross domestic product in advanced economies to more than 30% in South Asia and in the Middle East and North Africa [30]. Our study lends further support to the importance of women’s empowerment in politics and economy as these domains of the GGGI were strongly associated with climate adaptation, particularly readiness. However, education was the domain with the strongest association with climate adaptation, perhaps because it is a quintessential condition for women’s empowerment. Gender equality in the economic and political domains will never happen until women have fair access to education. Although in 2020 more than two-thirds of countries worldwide had reached gender parity (defined as having a gender parity index value between 0.97 and 1.03) in enrolment in primary education, gender disparities disadvantaging girls in primary education persisted in Africa, the Middle East and South Asia [31]. For instance, 78 girls in Chad and 84 girls in Pakistan were enrolled in primary school for every 100 boys in 2020 [31]. Unless women’s rights are respected and gender inequalities eliminated, our ability to adapt to climate change will be in jeopardy and, sadly, the worst consequences will be faced by women [1]. 

For the past years of international climate change negotiations, governments worldwide have agreed that promoting gender equality and protecting women’s rights are crucial to meet global climate and development goals. This was formally recognised in the Gender Action Plan, which was approved at COP25 and subsequently reviewed in more recent COP meetings. The plan sets out objectives and activities that aim to “advance knowledge and understanding of gender-responsive climate action and promote women’s full, equal, and meaningful participation” in the UNFCCC process. Notwithstanding some initiatives to enhance women’s participation, from travel funds to mentorship networks, progress remains uneven and unacceptably slow [32]. After reaching a peak of almost 40% at the Conference of the Parties (COP) 24, women’s representation in country delegations has been in decline [32]. For instance, at COP27, women delegates accounted for 36% of all national Party delegates, which is better than the 35% at the previous year but lower than pre-pandemic COPs. Likewise, at COP27 women only encompassed 20% of Heads of Delegation, revealing an increase from COP26 (13%) but a decrease from previous COPs. In some countries, men accounted for 90% of the delegations. This means that the voices of women are not being heard commensurately with the impact of climate change on their lives at the most visible global arena. The dire representation of women at the negotiating table raises questions about the true commitment of the parties to address the gendered impact of climate change. In addition, lack of gender-disaggregated data prevents a comprehensive evaluation of the gendered consequences of climate change and the development of gender specific policy and interventions [23]. Without fair representation of women, and evidence on the gender-specific impact of climate change, the commitments of the Gender Action Plan will continue to be unmet. Unless longstanding gender inequalities across several domains of society are uprooted, effective climate mitigation and adaptation targets will not be achieved, hence threatening the lives and livelihoods of women and men alike.

### Limitations

The robustness of the conclusions when using two different indices of gender equality, and the inclusion of the majority of the countries in the world, attest confidence to our findings. However, these findings need to be interpreted in light of methodological limitations. First, as an aetiological study, it is possible that the associations observed at country level do not reflect associations at a local level. Second, it is an observational, cross-sectional study and hence cannot prove causality. Third, data were not available for all countries in the world and including all countries could have changed some of the results. However, data were available for the majority of the countries, and it is unlikely that the overall associations would be changed substantially by adding a few countries. Fourth, although we used two differences indices, only moderately correlated, to measure gender equality, neither is exhaustive in its assessment of gender equality, for which there is no gold standard metric or index. It is possible, if unlikely, that including other dimensions would have changed the overall associations. To complement our research and address its limitations, further research is warranted to understand to what extent gender-sensitive policies and interventions contribute to climate adaptation in high- and low- and middle-income countries.

## Conclusions

Gender inequality, measured broadly across different domains of life, is associated with lack of climate adaptation at country level, both in terms of vulnerability to impact and readiness to respond. Therefore, promoting gender equality and empowering women is critical for countries to adapt to the increasing threats posed by poorly mitigated climate change, which will fall disproportionately on women.

### Supplementary Information

Supplementary Material 1.

## Data Availability

All data are available on the GitHub repository https://github.com/Ana-Catarina/Climate-and-gender.git.
