# Supplementary material for: The association between gender equality and climate adaptation across the globe
Source: BMC Public Health. 2024 May 24;24:1394. doi: 10.1186/s12889-024-18880-5 (PMC11127422; doi:10.1186/s12889-024-18880-5)

**Supplementary data**

**Table S1: Indicators included in the Global Gender Gap Index**

| Subindex | Indicator |
| --- | --- |
| Economic Participation and Opportunity | Labour force participation rate (%) |
|  | Wage equality for similar work (survey, 1–7 scale) |
|  | Estimated earned income (PPP, int.$) |
|  | Legislators, senior officials and managers (%) |
|  | Professional and technical workers (%) |
| Educational Attainment | Literacy rate (%) |
|  | Enrolment in primary education (%) |
|  | Enrolment in secondary education (%) |
|  | Enrolment in tertiary education (%) |
| Health and Survival | Sex ratio at birth (%) |
|  | Healthy life expectancy (years) |
| Political Empowerment | Women in parliament (%) |
|  | Women in ministerial positions (%) |
|  | Years with female head of state (last 50), share of tenure years |

**Table S2: Indicators included in the Gender Inequality Index**

| Dimension | Indicator |
| --- | --- |
| Health | Maternal mortality ratio |
|  | Adolescent birth rate |
| Empowerment | Female and male population with at least secondary education |
|  | Female and male shares of parliamentary seats |
| Labour Market | Female and male labour force participation rates |

**Table S3: Countries included in this study**

| Afghanistan | France | New Zealand |
| --- | --- | --- |
| Albania | Gambia | Nicaragua |
| Algeria | Georgia | Niger |
| Angola | Germany | Nigeria |
| Argentina | Ghana | North Macedonia |
| Armenia | Greece | Norway |
| Australia | Guatemala | Oman |
| Austria | Guinea | Pakistan |
| Azerbaijan | Guyana | Panama |
| Bahrain | Honduras | Paraguay |
| Bangladesh | Hungary | Peru |
| Barbados | Iceland | Philippines |
| Belarus | India | Poland |
| Belgium | Indonesia | Portugal |
| Belize | Iran | Qatar |
| Benin | Ireland | Romania |
| Bhutan | Israel | Rwanda |
| Bolivia | Italy | Saudi Arabia |
| Bosnia and Herzegovina | Jamaica | Senegal |
| Botswana | Japan | Serbia |
| Brazil | Jordan | Sierra Leone |
| Brunei Darussalam | Kazakhstan | Singapore |
| Bulgaria | Kenya | Slovakia |
| Burkina Faso | Kuwait | Slovenia |
| Burundi | Kyrgyzstan | South Africa |
| Cambodia | Laos | South Korea |
| Cameroon | Latvia | Spain |
| Canada | Lebanon | Sri Lanka |
| Cape Verde | Lesotho | Suriname |
| Chad | Liberia | Sweden |
| Chile | Lithuania | Switzerland |
| China | Luxembourg | Tajikistan |
| Colombia | Madagascar | Tanzania |
| Comoros | Malawi | Thailand |
| Costa Rica | Malaysia | Timor-Leste |
| Cote d'Ivoire | Maldives | Togo |
| Cyprus | Mali | Tunisia |
| Czech Republic | Malta | Turkey |
| Denmark | Mauritius | Uganda |
| Dominican Republic | Mexico | Ukraine |
| DR Congo | Moldova | United Arab Emirates |
| Ecuador | Mongolia | United Kingdom |
| Egypt | Montenegro | United States |
| El Salvador | Morocco | Uruguay |
| Estonia | Mozambique | Vanuatu |
| Eswatini | Myanmar | Vietnam |
| Ethiopia | Namibia | Zambia |
| Fiji | Nepal | Zimbabwe |
| Finland | Netherlands |  |

**Figure S1: World map illustrating the distribution of the vulnerability and readiness subindices of the Notre Dame Global Adaptation Index**


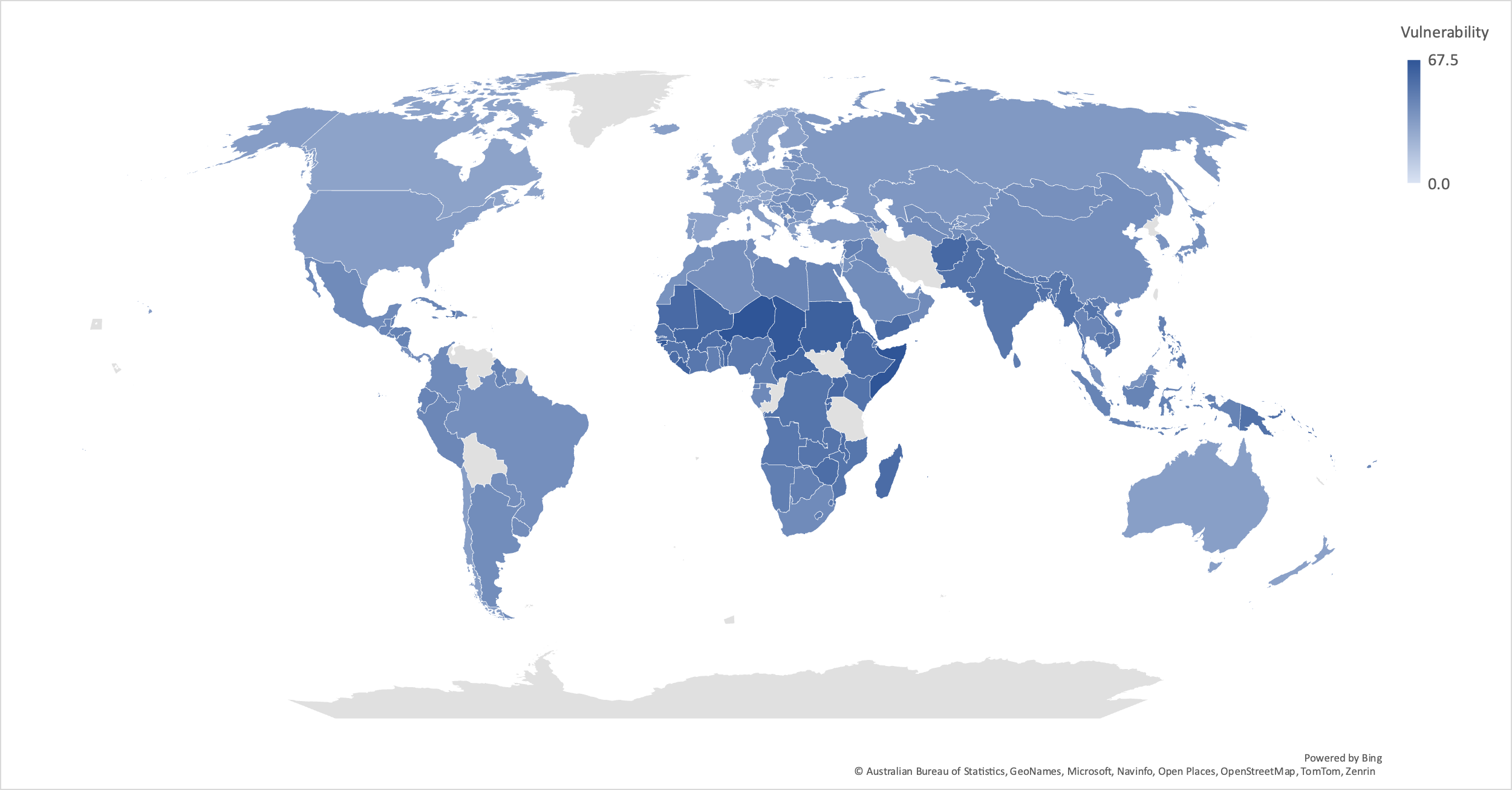


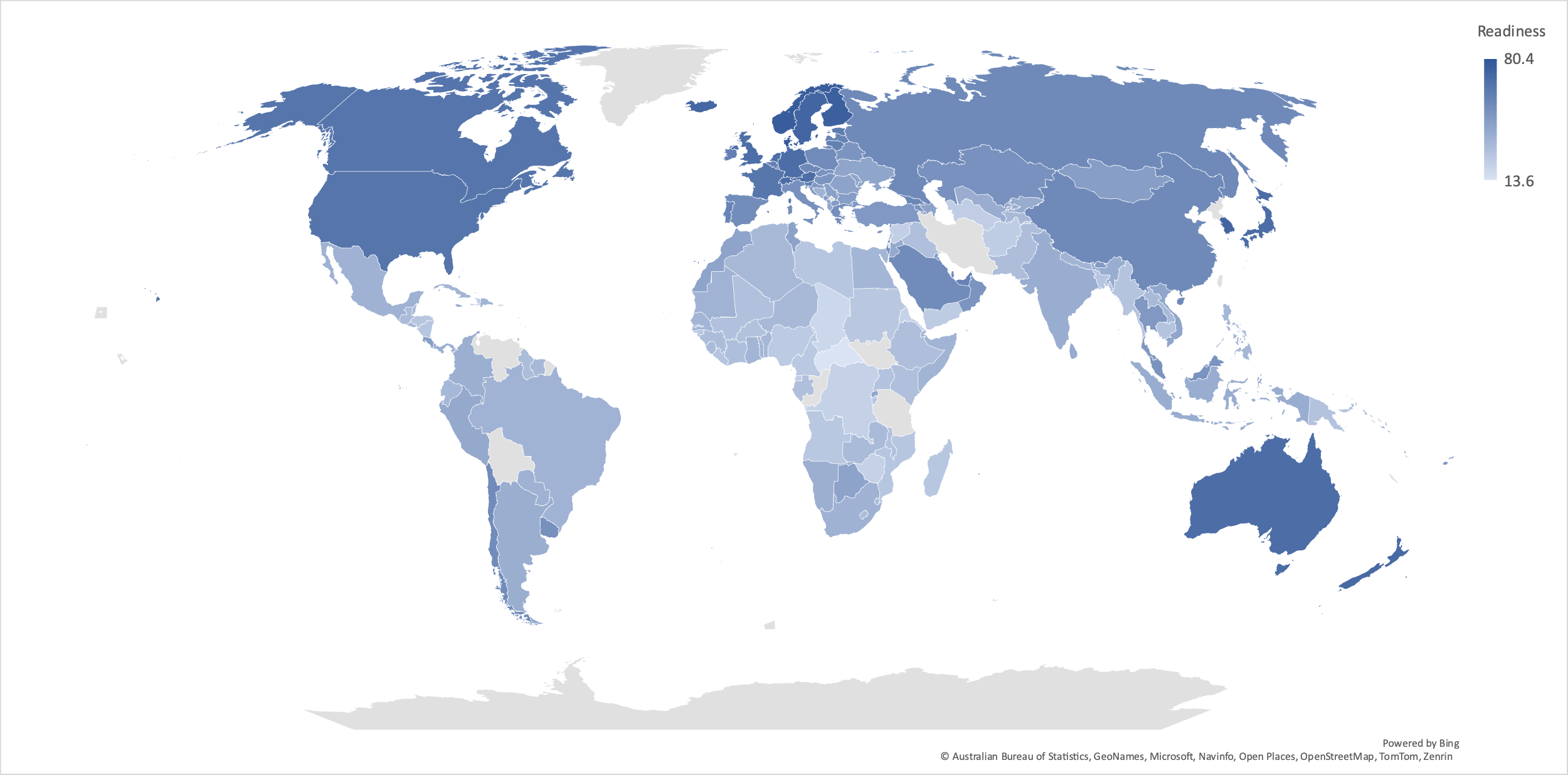

Supplement: Supplementary file 1 — Supplementary Material 1. [file 12889_2024_18880_MOESM1_ESM.docx]
